# Exploring Deep Learning Approaches for Walnut Phenotype Variety Classification

**DOI:** 10.1155/ijfo/9677985

**Published:** 2025-03-18

**Authors:** Burak Yılmaz

**Affiliations:** Faculty of Engineering and Natural Sciences, Department of Software Engineering, Konya Technical University, Konya, Türkiye

**Keywords:** classification, deep learning, InceptionV3, k-NN, logistic regression, SVM, VGG-16, VGG-19, walnut

## Abstract

The efficient classification of agricultural commodities like walnuts is crucial for assessing quality and managing the supply chain. This scholarly article analyses various deep learning and data science methods for walnut fruit classification. For this purpose, first, a dataset comprising images of walnuts from Chandler, Fernor, Howard, and Oguzlar varieties was collected. Two different experiments were conducted. In the first experiment, only deep learning methods were used as classifiers. In this experiment, InceptionV3 demonstrated the highest classification accuracy, followed by VGG-19 and VGG-16. In the second experiment, deep learning algorithms were used for feature extraction, followed by support vector machine (SVM), logistic regression (LR), and k-nearest neighbor (k-NN) algorithms for classification. These models resulted in an improvement in overall success rates. The most effective classification was achieved with the InceptionV3 and LR combination, achieving the highest success rate. These results highlight the efficacy of deep learning methodologies in swiftly and accurately classifying agricultural products based on visual information, indicating the potential to strengthen classification systems within the agricultural sector.

## 1. Introduction

The quality of agricultural products is of critical importance to ensure the sustainable success of agricultural businesses and to meet consumers' demands for higher product standards [1]. Various stakeholders in the agricultural product supply chain, including suppliers, processors, and quality inspection departments, must implement quality measures to ensure product quality and safety [2]. The assurance of quality and safety in agricultural goods across the complete food supply chain is of utmost importance for human well-being and underscores the necessity for a thorough monitoring framework [3]. Prominent agricultural organizations prioritize enhancing quality and safety control protocols to align with consumer expectations and safeguard the integrity of agricultural products [4]. Phenotype classification is an important method for maintaining the quality and health of the agricultural production line.

Walnut is one of the agricultural products that require rapid phenotype classification during processing. The nutritional values of walnuts vary significantly depending on the type of walnut. Different types of walnuts exhibit variations in terms of moisture content, oil content, fatty acid composition, protein, carbohydrate, and mineral content [5]. Different studies have highlighted the significance of classification based on morphological, biochemical, and molecular markers [6]. Techniques like low-field nuclear magnetic resonance (LFNMR) have been utilized to classify walnut varieties based on water and oil content, aiding in determining storage conditions [7]. Fatty acid profiling has been identified as a key indicator for distinguishing walnuts from different geographical locations, providing insights into quality assessments and possible adulterations in the market [8]. Implementing walnut classifying and screening production lines can enhance sorting efficiency, size, weight, and quality identification in walnut sorting processes [9]. Among these methodologies, “computer vision” and “machine learning” applications in agriculture are gaining interest due to their nondestructive evaluation capabilities and lower costs compared to traditional manual methods [10]. These technologies offer a way to efficiently analyze and monitor agricultural products without causing any damage, making them a valuable tool for enhancing productivity and quality control in the industry. In the literature, deep learning methods are widely used for phenotype prediction and classification across a broad spectrum. For instance, the classification of rice varieties can be achieved using artificial intelligence methods [11].

When the literature is examined, machine learning methods have been extensively researched in the classification of walnut phenotypes. Substantial advancements and valuable insights have been achieved within this domain. Diverse machine learning algorithms have been employed to effectively categorize different traits of walnuts. In various studies in the literature, it has been seen that artificial neural networks (ANNs), support vector machines (SVMs), and random forests (RFs), which are well-known methods, are used in developing classification models to distinguish walnuts with certain characteristics, especially shrunken kernels [12, 13]. These approaches have exhibited notable levels of accuracy, notably with SVM attaining a classification accuracy (CA) rate of 97% in the identification of walnuts with shriveled kernels [13].

Moreover, machine learning techniques have been instrumental in enhancing the precision (Prec) of phenotypic profiling in walnuts. By leveraging these methods, researchers have been able to improve the accuracy of suture strength phenotyping in walnuts, enabling the application of quantitative trait loci (QTL) mapping and genome-wide association studies (GWAS) to dissect the genetic basis of walnut traits [14]. Additionally, machine learning models, such as deep convolutional neural networks (CNNs) (DCNNs) and SVM, have been employed to automatically classify phenotypic traits in walnuts based on various characteristics, including appearance and coloration [15].

Furthermore, the integration of machine learning with advanced technologies like X-ray computed tomography has enabled the 3D characterization of walnut morphological traits, showcasing the versatility of machine learning in analyzing complex walnut structures [16]. Additionally, machine learning algorithms have been utilized in conjunction with hyperspectral imaging technology for nondestructive detection of moldy walnuts, achieving an impressive overall CA of 93%.

The application of machine learning methods in the classification of walnut phenotypes has proven to be a powerful tool in enhancing accuracy, efficiency, and Prec in phenotypic profiling. By leveraging a diverse range of machine learning algorithms and technologies, researchers have made significant strides in understanding and classifying walnut traits, paving the way for advancements in walnut cultivation and agricultural research. The fact that deep learning methods, which are among the machine learning methods, can directly receive images as input has caused deep learning methods to attract attention and be seen as advantageous in this regard.

Deep learning methods stand out as an important tool in determining plant phenotypes. These methods can help plant breeders quickly and accurately identify plant traits, diagnose diseases, and increase productivity [17, 18]. Therefore, deep learning methods are considered an important tool for maintaining quality and health in determining plant phenotypes. Deep learning methods enable image-based high-throughput phenotype analysis to identify plant phenotypes [19]. These methods can automatically identify plant traits and help plant breeders make informed decisions.

In the context of plant phenotyping, deep learning models have been successfully applied in various applications, such as weed species classification and disease detection [20]. Specifically, deep learning has been used to establish ANN models for screening differentially expressed genes in walnut endocarps during the hardening period, overcoming the limitations of traditional algorithms [21]. Li et al. [22] developed a YOLOv8-based method for fast classification of walnut material. In this study, it was reported that walnut classes were accurately separated using a lightweight and fast CNN model [22].

Similarly, in a study by Guo et al. [23], deep learning was combined with phenotypic data for walnut maturity levels and oil content estimation. This approach is important not only for classification but also for walnut quality estimation [23].

Working on a more specific problem such as the separation of walnut shell and kernel, Ni et al. optimized a deep learning model that provided high accuracy in walnut shell-kernel separation with machine vision [24]. It was observed that image processing and data preprocessing techniques increased model performance. Moreover, the backpropagation ANN classification model has been employed to identify regional variations in walnuts with a high mean accuracy of 89.7% [25]. Karadeniz et al. created a walnut dataset and achieved successful classification using VGG-16, with an accuracy of 85.52% on the original dataset and 90.55% on the augmented dataset [26]. Additionally, Chen et al. proposed a deep-learning model combining spatial attention and SE-network structures, achieving a test set accuracy of 92.2% for walnut kernel grading, and outperforming VGG-19 and EfficientNet [27]. These findings collectively demonstrate the success of deep learning methods, particularly VGG-16, VGG-19, and novel models, in accurately classifying walnut phenotypes with impressive accuracy rates. Machine learning methods, including deep learning, have been instrumental in developing classification models for authenticating walnuts from different regions worldwide [28].  Table 1 represents brief information about the mentioned studies.

The application of deep learning architectures such as InceptionV3, SqueezeNet, VGG-16, and VGG-19 in walnut image categorization has yielded promising results. Research indicates that employing pre-existing CNN architectures proves to be proficient in accurately categorizing walnut leaf visuals [26]. Additionally, studies with pretrained CNN models such as VGG-16, DenseNet, MobileNet, InceptionV3, ResNet50, and Xception have highlighted advances in image classification tasks [29]. Thus, the integration of these pre-existing architectures can substantially enhance the Prec and effectiveness of walnut image categorization tasks while offering valuable insights for individuals involved in the domains of computer vision and deep learning.

Overall, the integration of deep learning methods in the classification of walnut phenotypes holds significant potential for accurate and efficient phenotypic profiling. By leveraging the capabilities of deep ANNs and machine learning algorithms, researchers can enhance the CA and speed of identifying walnut phenotypes, contributing to advancements in agricultural research and walnut cultivation practices.

In this study, the effectiveness of various deep learning methods for classifying walnut species was evaluated. Two different approaches were tested for this purpose. The first approach involved generating features using deep learning methods and then identifying the most effective features. These features were subsequently used for classification with various machine learning methods. The second approach directly utilized various deep learning methods for classification.

The main reason for choosing InceptionV3, VGG-16, and VGG-19 models is their proven success in image classification tasks. InceptionV3 stands out with its ability to efficiently extract detailed features and its low computational cost due to its innovative architecture. VGG-16 and VGG-19 offer powerful feature extraction capabilities with their deeper but simpler structures and are known for their versatility in different classification tasks. These models were selected to explore the performance of different architectures on the walnut dataset in order to provide a balance between computational efficiency and accuracy.

Extracting features with CNN models and classifying these features with traditional machine learning methods allowed us to benefit from the strengths of both methods. CNNs are very good at detecting complex patterns and features that are difficult to extract manually from image data. By removing FC layers, the resulting features represent high-level abstractions of the dataset. These features increase CA and interpretability when used with classifiers such as LR, SVM, and k-NN. This hybrid approach allowed us to examine the dataset in detail and explore the performance of feature-based classification compared to end-to-end deep learning models.

When selecting machine learning models, our main criteria were CA, computational efficiency, and the ability to process feature vectors extracted by CNNs. LR was chosen due to its simplicity and robust performance on linearly separable data. SVM was chosen due to its ability to effectively process high-dimensional data and its success in achieving high accuracy. k-NN was included to evaluate its performance as a nonparametric method. These models were chosen to comparatively analyze the strengths and weaknesses of different algorithms in walnut species classification based on extracted features. Another purpose of choosing these methods in the study is to adapt this model to industrial applications after obtaining an effective classification model.

The key objectives of this study include the following:

1. Developing efficient classification methods using a dataset of walnut images.
2. Comparing direct deep learning models (InceptionV3, VGG-16, and VGG-19) against hybrid approaches that combine deep learning feature extraction with machine learning classifiers like LR, SVM, and k-NN.
3. Identifying the best-performing model to improve the accuracy and efficiency of walnut classification systems.

The organization of this study is as follows: In the second section, the dataset, performance metrics, cross-validation, and methods used in the study are described. In the third section, the experimental results obtained in the study are presented. In the final section, the experimental results are interpreted, and recommendations are provided.

## 2. Materials and Methods

### 2.1. Dataset

In this study, phenotypic classification was conducted by examining four different types of walnuts. The dataset comprised walnuts of the Chandler, Fernor, Howard, and Oguzlar varieties. General information about these walnut types is as follows.

Chandler walnuts are recognized for their favorable fatty acid composition, with high levels of polyunsaturated fatty acids (PUFAs) [30]. Chandler walnuts are characterized by specific phenological and pomological traits, such as nut dimensions larger than traditional Chandler varieties, light kernel color, and easy kernel removal [31].

Fernor walnuts exhibit specific characteristics based on various studies. In terms of sensory evaluation, Fernor walnuts were noted to have a rough shell, dark pellicle, and less brightness in the kernel, making them one of the least crispy cultivars [32].

The appearance features of the Howard-type walnut were studied in various contexts. Lampinen et al. conducted a trial on pruned and unpruned Howard walnut trees, observing canopy growth and nut quality characteristics [33].

Oguzlar-type walnut, as studied in various contexts, exhibits specific characteristics related to storage and quality. Research by Yalçin et al. highlights that Oguzlar-77 walnuts maintain their quality under 0°C storage conditions, emphasizing the importance of suitable storage conditions for preserving freshness (Yalçin et al.).

**Figure 1:**
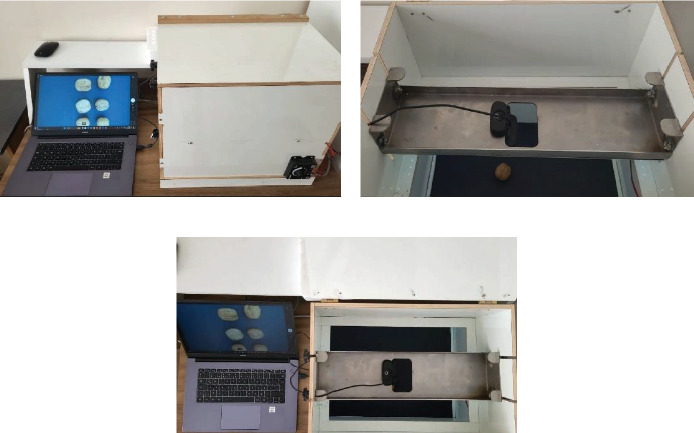
Image acquisition system. (a) System general view. (b) Camera setup of the system. (c) View from the upper section.

The dataset comprises various images of each class, captured using a specialized image acquisition system designed specifically for this type of task. The system consists of a sealed box equipped with dimmable internal lighting, a camera, and interchangeable backgrounds. For this study, a black background was utilized. The internal lighting was provided by 4000 K, 160 Lumen LED lights.  Figure 1 illustrates the image acquisition system.

Figure 1a represents the general view of the system, Figure 1b represents the camera system, which can be changed due to task, and Figure 1c represents the upper section of the system with images of walnuts captured by the computer. There are no moving parts in the mechanism. Walnuts were placed in various positions in front of the camera, and their images were captured.

**Figure 2:**
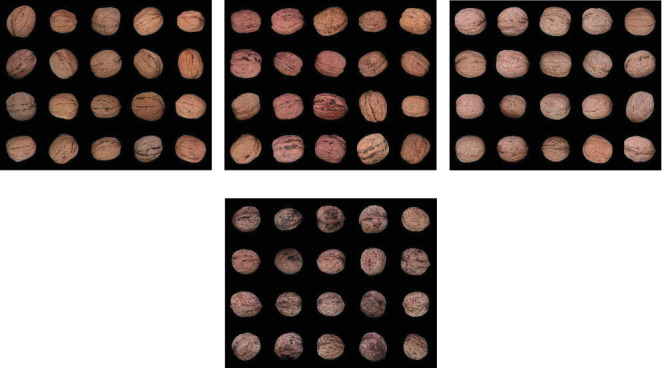
First 20 images from each class: (a) Chandler, (b) Fernor, (c) Howard, and (d) Oguzlar.

Images obtained from the image acquisition system were used to generate a dataset with four classes which are named Chandler, Fernor, Howard, and Oguzlar. While creating the dataset, each image was obtained separately. The images shown in Figure 2 are the first 20 images of each class in this dataset, combined into a single image.

In Figures 2, 2, 2, and 2, each class is shown as Chandler, Fernor, Howard, and Oguzlar. The dataset consists of 952 images.  Table 2 shows the number of images contained in each class in the dataset.

The dataset is used to train and test various machine learning models. In order to obtain a sufficient number of image data and to share the amount of data for each class in the same proportion as possible, a data set was created by selecting 3 kg of each walnut type.

### 2.2. Experiment Design

In this study, two different experimental approaches were adopted. In both approaches, deep learning models such as InceptionV3, VGG-16, and VGG-19 were preferred. InceptionV3, introduced by Szegedy et al. [34], is known for its efficiency in extracting detailed information from images while reducing training parameters. VGG-16 and VGG-19, on the other hand, are recognized for their simplicity and effectiveness in image classification tasks. The architectures examined in this study can be summarized as follows.

#### 2.2.1. VGG-Net Architecture

**Figure 3:**
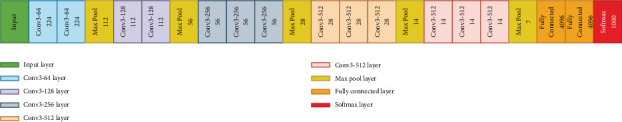
VGG-16 architecture.

VGG-Net, developed by the Visual Graphics Group in Oxford, made a breakthrough idea to reinforce the value of depth in deep ANNs. Featuring a simple architecture and stacking cascades of 3 × 3 convolution (Conv.) layers one after the other, VGG-Net models are readily interpretable and implementable. These characteristics have contributed to the popularity of VGG-Net in image feature extraction [35].  Figure 3 shows the VGG-16 architecture, which is characterized by sequential Conv. layers to capture hierarchical spatial features in walnut images. This structure plays a critical role in distinguishing walnut varieties in the dataset by effectively extracting visual patterns such as texture and shape.

**Figure 4:**
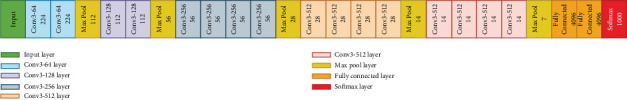
VGG-19 architecture.

VGG-16 and VGG-19 share a similar architecture, with the primary distinction being the number of layers. VGG-19, based on CNN principles, consists of 16 Conv. layers and 3 FC layers. These layers work together to transform image data into feature vectors.  Figure 4 presents the VGG-19 architecture, which is an extension of VGG-16. The added Conv. layers enhance the extraction of more complex features, especially subtle differences in walnut shell texture or kernel coloration, and improve the CA.

#### 2.2.2. InceptionV3 Architecture

**Figure 5:**
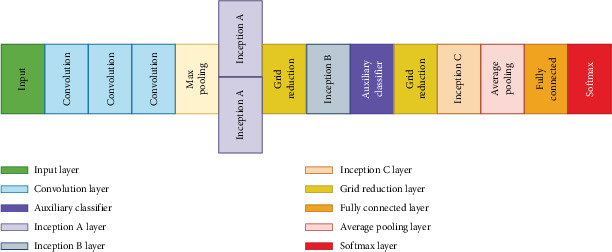
InceptionV3 architecture.

The architecture of InceptionV3, depicted in Figure 5, employs different filter sizes within the same Conv. block. This design enables the extraction of features at multiple scales from the input volume. For instance, it processes the input using 1 × 1, 2 × 2, 3 × 3, and 5 × 5 filters, and max-pooling (same padded) within a single block, resulting in feature volumes of varying depths. These volumes are then concatenated to produce a final output that is fed into the next layer. Computational complexity is reduced using 1 × 1 Conv., which decrease the channel depth of the volumes without altering their height and width. The simultaneous Conv. allow the network to expand in width. InceptionV3 further reduces computational complexity by factorizing Conv. into smaller and asymmetric operations [29]. Figure 5 shows the InceptionV3 architecture, which uses the multiscale approach. This architecture processes the inputs by passing walnut images through various filter sizes in a single Conv. layer. This design captures detailed features and broader contextual patterns, which increases the ability to distinguish walnut varieties.

The education of these models frequently includes extensive datasets like ImageNet, as referenced by [36]. For example, models such as InceptionV3 undergo training on millions of images and subsequently undergo assessment on validation sets to guarantee their Prec and generalization abilities. The process of training usually encompasses the enhancement of the models' parameters through methods such as backpropagation and gradient descent to reduce prediction inaccuracies.

**Figure 6:**
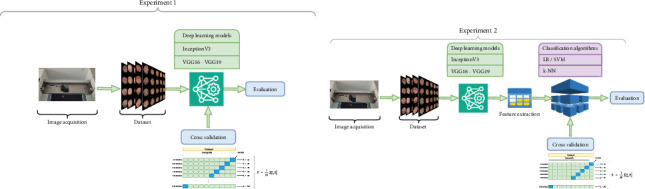
Experimental designs. (a) Only deep learning–based classification. (b) Deep learning features and classifier-based experiment.

In the first approach, the classification task was carried out only with deep learning approaches, and the “FC” layer was not removed in the last classification step. In the second approach, three deep learning models were used to extract deep learning features from the dataset. In this approach, the “FC” layer is removed from the models. Then, the features obtained with these models have been presented to classification algorithms which are LR, k-NN, and SVM.  Figure 6 represents both experimental designs simultaneously.

Figure 6 represents the experimental designs of the classification tasks.  Figure 6a represents the only deep learning-based classification Figure 6b represents the deep learning features and classifier-based classification. The classification algorithms used in the first can be explained as follows:

LR is one of the most widely used models. In LR, the independent variable is predicted using one or more independent variables. This method is used to combine arguments and independent variables. An important advantage of LR is that the variables are not normally distributed. Since predicted values are interpreted as probabilities, values of LR can be between 0 and 1. This is not a direct consequence of LR, but a consequence of estimating probability [11].

As a machine learning algorithm, k-NN is highly effective for classification tasks. Originally introduced by Fix and Hodges in 1951 [37], the method works by identifying *k* objects from the training set that closely match the test data. Based on the classes of these neighboring objects, the algorithm assigns the test data to the most common class among them [9].

SVM is a kernel-based method used for lights and regression. Thanks to its separation program, SVM can classify data as linear in two-dimensional space, plane in three-dimensional space, and hyperplane in multidimensional space. SVM involves the process of finding the best differentiating hyperplanes of data [13, 16].

### 2.3. Performance Metrics

Performance metrics are essential for evaluating the effectiveness of various systems and algorithms. The following performance metrics are used in this study. AUC (area under the curve): AUC represents the area under the receiver operating characteristic (ROC) curve, providing an overall measure of the model's CA. An AUC close to 0.5 indicates random predictions, while a score closer to 1 indicates strong classification performance. CA: This metric indicates the percentage of correctly classified instances out of the total instances. While it is a general measure of accuracy, it may be misleading on imbalanced datasets. *F*1 score: The F1 score is the harmonic means of Prec and recall, balancing both false positives and false negatives. It is particularly useful for evaluating performance on imbalanced datasets. Prec: Prec represents the proportion of positive predictions that are actually correct. High Prec indicates a low rate of false positives. Recall: This metric measures the model's ability to identify actual positives correctly. High recall suggests that the model has a low rate of false negatives.  Table 3 summarizes the performance metrics.

These metrics are crucial for assessing the performance of the models developed. They provide a comprehensive evaluation of a model's predictive accuracy and are vital for optimizing system performance.

### 2.4. Cross Validation

**Figure 7:**
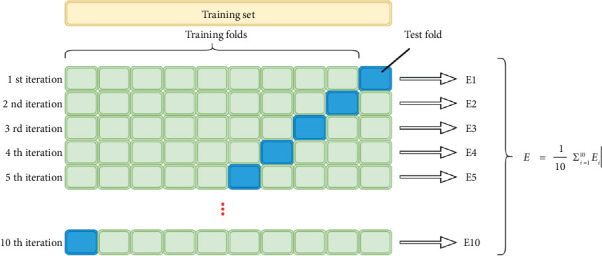
Cross-validation process.

Cross-validation is a fundamental technique in machine learning used to evaluate the performance and generalization ability of predictive models. It involves partitioning the dataset into subsets for training and testing, iteratively switching between different subsets for training and validation. This method helps in assessing how well a model will perform on unseen data, guarding against overfitting and providing a more accurate estimation of the model's predictive capabilities [38]. By repeating cross-validation, the variability in model performance due to different dataset splits can be accounted for, aiding in the selection of optimal models [38]. Additionally, cross-validation allows for the estimation of prediction errors, which is particularly useful when dealing with limited data, as it maximizes the use of available information [39]. It is a versatile tool that can be applied in various scenarios, even when precise likelihood derivation or parameter counting is challenging [40]. Moreover, cross-validation results in more stable evaluations compared to simply splitting data into training and validation sets [41].  Figure 7 represents the cross-validation process.

Overall, cross-validation is a crucial method in machine learning that ensures robust model evaluation and selection, significantly contributing to the reliability and generalizability of predictive models. In this study, each model is trained with the 10-fold cross-validation method.

## 3. Results

In the experiments carried out within the scope of this study, three different deep learning models (InceptionV3, VGG-19, and VGG-16) and the features extracted from these models were compared with various machine learning methods. In the first experiment, the direct classification performance of the models was evaluated and InceptionV3 stood out as the most successful model with a high AUC (0.995) and CA (0.947). In the second experiment, the “FC” layers of the deep learning models were removed and used as a feature extraction tool, and these features were classified with logistic regression (LR), SVM, and k-NN algorithms. The LR method showed superior performance in both accuracy and other metrics (CA: 0.951–0.961). In addition, the errors and correct predictions in the classification process were detailed by analyzing the confusion matrices. The results reveal the potential of the proposed approaches to increase CA and the opportunities for future studies to eliminate the deficiencies.

The parameters of the deep learning algorithms used in all experiments are listed in Table 4. The table provides a comparative overview of the three deep learning models used in the experiments, InceptionV3, VGG-16, and VGG-19. The table includes the basic features such as the number of layers in the architecture of the models, the total number of parameters (which determines the complexity of the model), and the input size they are designed to process. All models use the ReLU activation function for nonlinear transformations and support optimization techniques such as Adam. In addition, these models are pretrained on the ImageNet dataset and are quite effective for transfer learning in image recognition tasks. Structural differences such as the number of layers and parameters affect the performance and computational requirements of these models, providing flexibility for different usage scenarios.

In Table 4, Conv. are abbreviated as FC layers. The number of layers, parameter sizes, input sizes, and optimization methods of the models were taken from the sources that describe the original model designs. In addition, the information that these models were pretrained on the ImageNet dataset was included in the table as a basic feature commonly mentioned in the literature.

### 3.1. Experiment 1

In the first experiment, three deep learning methods were compared with the walnut dataset. An experiment was conducted with 10-fold cross-validation. The results of the classification process are shown in Table 5.

The performance metrics of three distinct deep learning methodologies, namely, InceptionV3, VGG-19, and VGG-16, are detailed in Table 5. The selected evaluation criteria encompass AUC, CA, *F*1 score (*F*1), Prec, and recall (Recall). InceptionV3 exhibits the highest AUC score of 0.995, followed closely by VGG-16 at 0.992 and VGG-19 at 0.988. Regarding overall CA, InceptionV3 emerges as the frontrunner with CA values of 0.947, trailed closely by VGG-19 and VGG-16 with CA values of 0.944 and 0.939, respectively. Furthermore, these models showcase commensurate performance across F1 score, Prec, and recall, with scores spanning from 0.939 to 0.948. These findings collectively underscore the robust performance of these deep learning methodologies across diverse evaluation metrics, accentuating their efficacy in classification endeavors.

Table 6 compares actual versus predicted classifications for four categories: Chandler, Fernor, Howard, and Oguzlar. Most instances are correctly classified, with 219 Chandler, 239 Fernor, 226 Howard, and 218 Oguzlar accurately identified. Misclassifications are relatively few, such as 8 Chandler instances mistaken for Fernor and 11 Fernor instances mistaken for Chandler. The total numbers are consistent, with 238 instances predicted as Chandler, 252 as Fernor, 240 as Howard, and 222 as Oguzlar, adding up to 952 instances in total. Overall, the classification model demonstrates high accuracy in predicting the correct categories, reflecting its effectiveness.

Table 7 represents the confusion matrix of classification performed by the VGG-19 method. The confusion matrix for the VGG-19 classifier reveals its performance in classifying four categories: Chandler, Fernor, Howard, and Oguzlar. Most instances are correctly classified, with 218 Chandler, 234 Fernor, 228 Howard, and 219 Oguzlar accurately identified. However, there are some misclassifications, such as 18 Fernor instances incorrectly predicted as Chandler and 8 Chandler instances misclassified as both Fernor and Howard. The totals are consistent, with 243 instances predicted as Chandler, 247 as Fernor, 242 as Howard, and 220 as Oguzlar, summing up to 952 instances overall. This indicates that the VGG-19 classifier is generally effective, demonstrating high accuracy in its predictions.

Table 8 represents the confusion matrix of classification performed by the VGG-16 method. The confusion matrix provided in Table 8 depicts the predicted versus actual classifications for four categories: Chandler, Fernor, Howard, and Oguzlar. It demonstrates the number of instances classified correctly and misclassified for each category. Notably, Chandler has 220 correct predictions, while Fernor has 236 correct predictions. Howard has 220 correct predictions, and Oguzlar has 218 correct predictions. However, there are misclassifications, such as 15 Fernor instances incorrectly classified as Chandler and 10 Howard instances misclassified as Chandler. The overall totals for each category are consistent, with 246 instances predicted as Chandler, 249 as Fernor, 236 as Howard, and 221 as Oguzlar, summing up to 952 instances in total. This suggests that while the model generally performs well, there are some misclassifications that need to be addressed for improved accuracy.

### 3.2. Experiment 2

In the second experiment, three deep learning methods were compared with the walnut dataset. However, unlike the first experiment, in this experiment, the “FC” layers in the deep learning methods were removed. In the second experiment, deep learning methods were used for feature extraction. These features obtained from the deep learning methods were classified with “LR,” “SVM,” and “k-NN” methods. The experiment was performed with 10-fold cross-validation. The parameters of these machine learning models are represented in Tables 9, 10, and 11. These parameters are commonly used parameters according to the literature.

Experiment 2 was conducted with these three machine learning models, with the parameters mentioned in Tables 9, 10, and 11. The parameters were selected to be as standard as possible. In addition, since the number of parameters produced by deep learning methods, especially when used as an extraction mechanism, is high, hence parameters such as the number of leaves, nodes, or neighborhoods were selected at higher values than the standard values.

#### 3.2.1. Experiment 2-A (VGG-16 as Feature Extraction Method)

In Experiment 2-A, the VGG-16 model was used as a feature extraction method. By applying this approach, each image in the dataset has been encoded as a data vector with 1024 features by the VGG-16. Then, the new feature-based data set has been classified with the LR, SVM, and k-NN algorithms. The results of the Experiment 2-A are represented in Table 12.

As illustrated in Table 12, LR showcases the highest AUC score of 0.995 and emerges as the top performer in overall CA, boasting a CA of 0.951. Following closely behind is SVM, which presents an AUC of 0.992 and a CA of 0.939. Conversely, k-NN displays slightly inferior performance metrics, recording an AUC of 0.986 and a CA of 0.896. These findings collectively emphasize the efficacy of these machine learning models in classification endeavors, notably with LR standing out for its superior performance in this assessment. Since the LR method gives the most successful results, we can understand how much of the data set it classifies correctly by looking at the confusion matrix of the method.  Table 13 shows this confusion matrix of Experiment 2-A.

The confusion matrix provided depicts a comparison between the expected and observed categorizations for four distinct groups: Chandler, Fernor, Howard, and Oguzlar. It reveals the exact number of correctly and incorrectly classified occurrences within each group. Specifically, Chandler is linked to 220 correctly predicted cases, Fernor with 240, Howard with 226, and Oguzlar with 219. Nevertheless, there are misclassifications present, such as 11 cases of Fernor inaccurately labeled as Chandler and 8 cases of Howard misclassified as Chandler. The total forecasts for each group show consistency, with 239 forecasts for Chandler, 251 for Fernor, 242 for Howard, and 220 for Oguzlar, totaling 952 forecasts. These findings suggest an overall effective categorization process, though there is room for improvement in reducing misclassifications to enhance accuracy.

#### 3.2.2. Experiment 2-B (VGG-19 as Feature Extraction Method)

In Experiment 2-B, the VGG-19 model was used as the feature extraction method. By applying this approach, each image in the dataset has been encoded as a data vector with 1024 features by the VGG-16. Then, the new feature-based data set has been classified with the LR, SVM, and k-NN algorithms. The results of the Experiment 2-A are represented in Table 14.

As shown in Table 10, LR attains the highest AUC value of 0.993 and excels in overall CA by achieving a CA of 0.951. In comparison, SVM closely follows with an AUC of 0.990 and a CA of 0.928. Conversely, k-NN exhibits relatively lower performance metrics, registering an AUC of 0.978 and a CA of 0.875. These findings imply that LR and SVM prove to be notably efficient in classification tasks, whereas k-NN also delivers commendable performance. These findings collectively underscore the effectiveness of the machine learning models in classification tasks, particularly highlighting LR for its exceptional performance in this evaluation. The superior outcomes yielded by the LR approach enable a comprehensive understanding of its accurate classification of the dataset through examination of the method's confusion matrix. The confusion matrix for Experiment 2-B is presented in Table 15.

The presented confusion matrix in Table 11 depicts the comparison between the anticipated and actual categorizations across four distinct groups: Chandler, Fernor, Howard, and Oguzlar. Specifically, Chandler is associated with 218 accurately predicted instances, whereas Fernor, Howard, and Oguzlar are linked to 236, 231, and 220 correctly predicted instances, respectively. Nevertheless, there exist misclassifications within the data, such as 16 instances of Fernor incorrectly identified as Chandler and 4 instances of Howard erroneously classified as Chandler. The total number of projections for each category remains constant, with 239 projections for Chandler, 248 for Fernor, 245 for Howard, and 220 for Oguzlar, resulting in an aggregate of 952 instances. These observations imply a generally proficient classification system, albeit with opportunities to refine the process to reduce misclassifications and enhance Prec.

#### 3.2.3. Experiment 2-C (InceptionV3 as Feature Extraction Method)

Experiment 2-C utilized the InceptionV3 model for feature extraction. This method involved encoding each image in the dataset into a data vector consisting of 1000 features generated by the InceptionV3 model. Subsequently, the dataset based on these new features was subjected to classification using LR, SVM, and k-NN algorithms. The outcomes of Experiment 2-C can be observed in Table 16.

As shown in Table 16, LR and SVM showcase analogous performance levels, achieving an AUC of 0.995, thereby highlighting their high accuracy with CA values of 0.961 and 0.953, correspondingly. k-NN also delivers commendable results, securing an AUC of 0.985 and a CA of 0.903. These findings underscore the efficacy of these machine learning approaches in making precise classifications. These results collectively emphasize the efficacy of machine learning models in classification assignments, with a specific focus on LR which stands out for its outstanding performance in this assessment. The excellent results produced by the LR technique facilitate a thorough comprehension of its precise classification of the dataset by analyzing the method's confusion matrix. The confusion matrix corresponding to Experiment 2-C is displayed in Table 17.

The confusion matrix in Table 17 delineates the anticipated as opposed to actual categorizations across four distinct classes: Chandler, Fernor, Howard, and Oguzlar. It delineates the count of accurately classified and erroneously classified occurrences within each specific category. Chandler is associated with 224 accurately predicted instances, Fernor with 243, Howard with 228, and Oguzlar with 220. Nonetheless, inaccuracies arise, exemplified by 9 Fernor instances being inaccurately categorized as Chandler and 7 Howard instances misclassified as Chandler. The cumulative forecasts for each category remain uniform, with 240 instances foreseen as Chandler, 251 as Fernor, 238 as Howard, and 223 as Oguzlar, culminating in 952 instances in total. These results imply proficient categorization overall, although there exists potential for refining accuracy by reducing misclassifications.

## 4. Discussion

It is of great importance to classify agricultural products during production to evaluate them, ship them correctly, and protect the supply chain. Classifying walnuts in this way provides a great advantage in the production and packaging process. Classification methods have been developed by looking at the leaves of the trees while they are still growing before the fruits are collected [26], and these methods are very successful. In addition, there is a need for rapid classification during packaging on the production line. In this context, a method is needed to classify the fruits of walnuts.

After obtaining the image of the walnut fruit, it is possible to extract various features and then classify and analyze them. Apart from this, another method is to classify it directly with deep learning methods or to classify it by presenting the features obtained from deep learning methods to classification algorithms.

In this study, a deep learning-based classification method was developed to classify walnut fruits. In the beginning, four types of walnut images were obtained with a special image-obtaining system with inner lightning. The walnut types were named Chandler, Fernor, Howard, and Oguzlar. By using these images, a dataset has been generated. The dataset consists of 952 images.

In the first experiment, this dataset was used to train and test three different deep learning methods, which are InceptionV3, VGG-19, and VGG-16. Considering the CA values, InceptionV3 showed the highest success with 0.947, while VGG-19 and VGG-16 reached CA values of 0.944 and 0.939, respectively.

In the second experiment, InceptionV3, VGG-19, and VGG-16 are used as feature extraction algorithms. The features obtained by these methods represented the LR, SVM, and k-nearest neighbor (k-NN) algorithms. It has been observed that overall success rates increase with this approach. Among these methods, LR was the most successful method in all trials. The most successful combination was obtained with the combination of InceptionV3 and LR methods. Considering the CA values, VGG-16 + LR achieved 0.951, VGG-19 + LR achieved 0.951, and InceptionV3 + LR achieved 0.961 success rates.

The results show that deep learning-based classification methods serve as effective methods that can be used to develop a classification system using images.  Table 18 represents the results in a tabular format to provide an immediate understanding of model performance.

**Figure 8:**
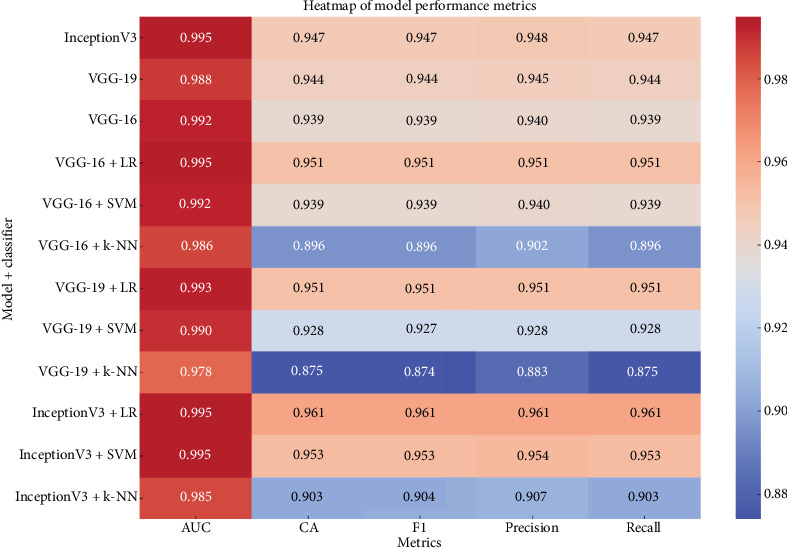
Heatmap of model performance metrics.

The heatmap graph in Figure 8 provides a comparative view of the metrics for all models and classifiers. It highlights the superior performance of the InceptionV3 + LR combination, achieving the highest CA of 0.961.

As can be seen in Figure 8, the combination of InceptionV3 and LR achieved the highest success in all metrics and offers great potential for increasing automation in the agricultural sector. This method can contribute to economic and environmental sustainability while increasing the efficiency of agricultural processes.

## 5. Conclusion

This study investigated the application of deep learning methodologies for the classification of walnut varieties based on phenotype images. The research is aimed at enhancing the accuracy and efficiency of automated classification systems, which hold significant potential for agricultural production, quality control, and supply chain management. Two experimental approaches were employed: (i) direct deep learning–based classification using CNN architectures and (ii) feature extraction using deep learning models, followed by classification with traditional machine learning algorithms.

The first set of experiments demonstrated that the InceptionV3 model achieved the highest CA (94.7%) among the three tested CNN architectures (InceptionV3, VGG-16, and VGG-19). The second experimental approach, in which features were extracted using deep learning models and then classified using LR, SVM, and k-NN, resulted in improved classification performance. The best-performing model was InceptionV3 combined with LR, achieving a CA of 96.1%, the highest recorded in this study.

The findings emphasize the effectiveness of deep learning models in agricultural classification tasks, particularly when combined with traditional machine learning techniques. The results also suggest that integrating CNN-extracted features with conventional classifiers can enhance accuracy and computational efficiency, making the approach suitable for industrial applications. The successful classification of walnut varieties using deep learning could facilitate automated sorting, improved quality control, and more efficient agricultural processing pipelines.

Future research directions may include (i) expanding the dataset to include a broader range of walnut varieties, (ii) implementing real-time classification on production lines using edge computing or IoT-based solutions, and (iii) incorporating additional data types, such as spectral or biochemical properties, to further refine CA. The integration of AI-driven classification models into agricultural workflows has the potential to significantly improve automation, reduce manual labor, and ensure consistency in walnut grading and distribution.

## Figures and Tables

**Table 1:** Similar studies in the literature.

**Reference**	**Article title**	**Focus**	**Method/technology**	**Success rate**
Shah et al. [5]	*Bio-Chemical Composition of Some Walnut (Juglans regia L.) Genotypes of North-Western Himalayan Region*	Nutritional and biochemical variations in walnuts	Morphological, biochemical, and molecular markers	Not applicable
Song et al. [7]	*Classification of Different Walnut Varieties Using Low-Field Nuclear Magnetic Resonance Technology and Cluster Analysis*	Classification based on water and oil content using LFNMR technology	Low-field nuclear magnetic resonance (LFNMR)	Not applicable
Pei et al. [8]	*Fatty Acid Profiling in Kernels Coupled with Chemometric Analyses as a Feasible Strategy for the Discrimination of Different Walnuts*	Quality assessment of walnuts from different regions	Fatty acid profiling	Not applicable
Karadeniz et al. [9]	*Classification of Walnut Dataset by Selecting CNN Features with Whale Optimization Algorithm*	Efficient walnut variety classification using CNN and optimization	VGG-16 with whale optimization algorithm	90.55% (augmented dataset)
Cinar and Koklu [11]	*Classification of Rice Varieties Using Artificial Intelligence Methods*	Rice variety classification	Artificial neural networks (ANN)	94%
Zhai et al. [13]	*Machine Learning for Detection of Walnuts with Shriveled Kernels by Fusing Weight and Image Information*	Walnut kernel classification with machine learning	Weight and image data fusion, SVM	97%
Xu et al. [16]	*Non-Destructive Detection of Moldy Walnuts Based on Hyperspectral Imaging Technology*	Moldy walnut detection using hyperspectral imaging	Hyperspectral imaging with machine learning	93%
Hosseini et al. [14]	*Efficient Phenotypic Sex Classification of Zebrafish Using Machine Learning Methods*	Machine learning in walnut phenotyping	Genome-wide association studies, QTL mapping	100%
Guo et al. [23]	*Phenotypic-Based Maturity Detection and Oil Content Prediction in Xiangling Walnuts*	Walnut maturity and oil content estimation using deep learning	Phenotypic-based analysis	Not applicable
Hridoy et al. [18]	*Improved Vision-Based Diagnosis of Multi-Plant Disease Using an Ensemble of Deep Learning Methods*	Multi-plant disease detection	Deep learning	99.20%
G. Chen et al. [19]	*Application of Plant Phenotype Extraction Using Virtual Data with Deep Learning*	High-throughput phenotype analysis	Image-based phenotype analysis	94%
Olsen et al. [20]	*Weed Species Classification and Disease Detection*	Weed classification and disease detection	Deep learning	95.7%
Z. Guo et al. [21]	*Screening and Functional Prediction of Differentially Expressed Genes in Walnut Endocarps*	Walnut endocarp gene expression analysis	Neural network models	AUC = 0.9796
Gu et al. [25]	*Multisource Fingerprinting for Region Identification of Walnuts in Xinjiang*	Regional identification of walnuts	Backpropagation neural network classification model	89.7%
S. Chen et al. [27]	*Effectiveness of VGG-19 in Disease Prediction*	Disease classification using transfer learning	VGG-19, transfer learning	94%
Segelke et al. [28]	*Developing Classification Models for Authenticating Walnuts*	Walnut authentication	Machine learning, deep learning	94%

**Table 2:** Amount of images contained in each class in the dataset.

**Total**	**Chandler**	**Fernor**	**Howard**	**Oguzlar**
952	234	258	238	222

**Table 3:** Performance metrics.

**Metrics**	**Formula**	**Evaluation description**
AUC (area under curve)	Area under ROC curve	Measures the classification accuracy of the model. Values close to 1 indicate strong performance, values close to 0.5 indicate random predictions.
CA (classification accuracy)	(TP + TN)/(TP + FP + TN + FN)	The ratio of correctly classified examples to the total examples. Can be misleading in imbalanced datasets.
F1 score	2^∗^ (Precision^∗^ Recall)/(Precision + Recall)	The harmonic mean of precision and sensitivity. Used to evaluate performance in imbalanced datasets.
Prec (precision)	TP/(TP + FP)	The ratio of true positive predictions to total positive predictions. High precision indicates low false positive rate.
Recall	TP/(TP + FN)	Measures the ratio of true positive examples to be correctly predicted. High sensitivity indicates low false negative rate.

**Table 4:** Parameters of deep learning models used in all experiments.

**Model**	**Number of layers**	**Number of parameters**	**Input size**	**Activation function**	**Optimization**	**Pretrained weights**
InceptionV3	48 (inception modules)	23 million	299 × 299 pixels RGB	ReLU	Adam	ImageNet
VGG-16	16 (13 Conv. + 3 FC)	138 million	224 × 224 pixels RGB	ReLU	Adam	ImageNet
VGG-19	19 (16 Conv. +3 FC)	144 million	224 × 224 pixels RGB	ReLU	Adam	ImageNet

**Table 5:** Performance metrics of deep learning methods.

**Model**	**AUC**	**CA**	**F1**	**Prec**	**Recall**
InceptionV3	0.995	0.947	0.947	0.948	0.947
VGG-19	0.988	0.944	0.944	0.945	0.944
VGG-16	0.992	0.939	0.939	0.940	0.939

**Table 6:** Confusion matrix of InceptionV3 classifier.

		**Predicted**				
		**Chandler**	**Fernor**	**Howard**	**Oguzlar**	∑
Actual	Chandler	219	8	7	0	234
	Fernor	11	239	6	2	258
	Howard	8	2	226	2	238
	Oguzlar	0	3	1	218	222
	∑	238	252	240	222	952

**Table 7:** The confusion matrix of the VGG-19 classifier.

		**Predicted**				
		**Chandler**	**Fernor**	**Howard**	**Oguzlar**	∑
Actual	Chandler	218	8	8	0	234
	Fernor	18	234	6	0	258
	Howard	5	4	228	1	238
	Oguzlar	2	1	0	219	222
	∑	243	247	242	220	952

**Table 8:** The confusion matrix of the VGG-16 classifier.

		**Predicted**				
		**Chandler**	**Fernor**	**Howard**	**Oguzlar**	∑
Actual	Chandler	220	7	7	0	234
	Fernor	15	236	7	0	258
	Howard	10	5	220	3	238
	Oguzlar	1	1	2	218	222
	∑	246	249	236	221	952

**Table 9:** Parameters of support vector machine (SVM).

**Parameter**	**Description**	**Value**
Kernel	Type of kernel function modeling relationships between functions	RBF exp(−auto|*x* − *y*|^2^)
C (regularization)	Regularization parameter. Low C tolerates errors; high C reduces them	1.0
Gamma	Scaling parameters are used for RBF, poly, or sigmoid kernels	Auto
Max iterations	Maximum number of iterations during training	100

**Table 10:** Parameters of logistic regression (LR).

**Parameter**	**Description**	**Common values**
Penalty	Type of regularization	L2
C (strenght)	Parameter controlling regularization strength Smaller values mean stronger regularization	1.0
Max iterations	Maximum number of iterations during training	100
Multiclass	Strategy used for multiclass tasks	ovr (one-vs.-rest)

**Table 11:** Parameters of k-nearest neighbors (k-NNs).

**Parameter**	**Description**	**Value**
n_neighbors	Number of nearest neighbors	50
Weights	Type of weights assigned to neighbors	Distance
Algorithm	Algorithm used for finding neighbors	Auto
Leaf size	Leaf node size used for tree-based algorithms	30
Metric	Distance measurement metric	Euclidean
*p*	Power parameter for Minkowski metric (1 = Manhattan; 2 = Euclidean)	2

**Table 12:** Results of Experiment 2-A (VGG-16 as feature extraction method).

**Model**	**AUC**	**CA**	**F1**	**Prec**	**Recall**
Logistic regression	0.995	0.951	0.951	0.951	0.951
SVM	0.992	0.939	0.939	0.940	0.939
k-NN	0.986	0.896	0.896	0.902	0.896

**Table 13:** The confusion matrix for Experiment 2-A.

		**Predicted**				
		**Chandler**	**Fernor**	**Howard**	**Oguzlar**	∑
Actual	Chandler	220	7	7	0	234
	Fernor	11	240	7	0	258
	Howard	8	3	226	1	238
	Oguzlar	0	1	2	219	222
	∑	239	251	242	220	952

**Table 14:** Results of Experiment 2-B (VGG-19 as feature extraction method).

**Model**	**AUC**	**CA**	**F1**	**Prec**	**Recall**
Logistic regression	0.993	0.951	0.951	0.951	0.951
SVM	0.990	0.928	0.927	0.928	0.928
k-NN	0.978	0.875	0.874	0.883	0.875

**Table 15:** The confusion matrix for Experiment 2-B.

		**Predicted**				
		**Chandler**	**Fernor**	**Howard**	**Oguzlar**	∑
Actual	Chandler	218	9	7	0	234
	Fernor	16	236	6	0	258
	Howard	4	3	231	0	238
	Oguzlar	1	0	1	220	222
	∑	239	248	245	220	952

**Table 16:** Results of Experiment 2-C (InceptionV3 as feature extraction method).

**Model**	**AUC**	**CA**	**F1**	**Prec**	**Recall**
Logistic regression	0.995	0.961	0.961	0.961	0.961
SVM	0.995	0.953	0.953	0.954	0.953
k-NN	0.985	0.903	0.904	0.907	0.903

**Table 17:** The confusion matrix for Experiment 2-C.

		**Predicted**				
		**Chandler**	**Fernor**	**Howard**	**Oguzlar**	∑
Actual	Chandler	224	4	6	0	234
	Fernor	9	243	4	2	258
	Howard	7	2	228	1	238
	Oguzlar	0	2	0	220	222
	∑	240	251	238	223	952

**Table 18:** The results of model performances.

**Model + classifier**	**AUC**	**CA**	**F1**	**Precision**	**Recall**
InceptionV3	0.995	0.947	0.947	0.948	0.947
VGG-19	0.988	0.944	0.944	0.945	0.944
VGG-16	0.992	0.939	0.939	0.094	0.939
VGG-16 + LR	0.995	0.951	0.951	0.951	0.951
VGG-16 + SVM	0.992	0.939	0.939	0.094	0.939
VGG-16 + k-NN	0.986	0.896	0.896	0.902	0.896
VGG-19 + LR	0.993	0.951	0.951	0.951	0.951
VGG-19 + SVM	0.099	0.928	0.927	0.928	0.928
VGG-19 + k-NN	0.978	0.875	0.874	0.883	0.875
InceptionV3 + LR	0.995	0.961	0.961	0.961	0.961
InceptionV3 + SVM	0.995	0.953	0.953	0.954	0.953
InceptionV3 + k-NN	0.985	0.903	0.904	0.907	0.903

## Data Availability

The data that support the findings of this study are openly available at http://muratkoklu.com/datasets/Walnut_4Class_Image_Dataset.zip.
